# Molecular iodine inhibits the expression of stemness markers on cancer stem-like cells of established cell lines derived from cervical cancer

**DOI:** 10.1186/s12885-018-4824-5

**Published:** 2018-09-26

**Authors:** Gabriele Davide Bigoni-Ordóñez, Elizabeth Ortiz-Sánchez, Pedro Rosendo-Chalma, Heriberto A Valencia-González, Carmen Aceves, Alejandro García-Carrancá

**Affiliations:** 1 División de Investigación Básica, Laboratory of Virus and Cancer, Instituto Nacional de Cancerología, Secretaria de Salud, Av. San Fernando No. 22, Sección XVI, Tlalpan, 14080 Ciudad de México, CP Mexico; 2 Programa de Maestría y Doctorado en Ciencias Bioquímicas, Facultad de Química, UNAM, Mexico City, Mexico; 3 Programa de Doctorado en Ciencias Biomédicas, UNAM, Mexico City, Mexico; 4 Instituto de Neurobiología, Universidad Nacional Autónoma de México, Boulevard Juriquilla 3001, Juriquilla. Campus-Juriquilla., Querétaro, 76230 Qro Mexico; 5 Instituto de Investigaciones Biomédicas, Universidad Naciona Autónoma de México, Mexico City, Mexico

**Keywords:** Molecular iodine, Cervical cancer stem cells, Stemness markers, PPAR gamma

## Abstract

**Background:**

Cancer stem cells (CSC) are characterized by deregulated self-renewal, tumorigenicity, metastatic potential, aberrant stemness signaling pathways, resistance to conventional therapy, and the ability to give rise to a progeny of proliferating cells that constitute the bulk of tumors. Targeting CSC will provide novel treatments for cancer. Different investigations have focused on developing complementary approaches that involve natural compounds that decrease chemo-resistance and reduce the side effects of conventional therapies. Since, it has been reported that molecular iodine (I_2_) exhibits antineoplastic effects and decreases tumor progression in some cancer models, we evaluated the potential effect of I_2_ on cell cultures enriched in cervical cancer stem-like cells.

**Methods:**

HeLa and SiHa cervical cancer cells were treated with 200uM I_2_ for 24 h. After time, cells were cultured in CSC-conditioned medium (cervospheres) and viability assays were performed. Following, tumorigenic capabilities in cervospheres treated with I_2_ were evaluated in NOD/SCID mice. HeLa monolayer cells untreated and their respective cervosphere cells treated or untreated with 200 μM of I_2_ for 24 h were xenotransplanted subcutaneously at different amounts and mice were monitored for at least 2 months.

**Results:**

In the present study, monolayer and CSC-enriched cultures (cervospheres) from cervical cancer-derived cell lines, HeLa and SiHa, showed that 200uM I_2_ supplementation inhibits proliferation of both and decreased their tumorigenic capacity, in vivo. This antineoplastic effect of I_2_ was accompanied by diminished expression of stemness markers including CD49f, CK17, OCT-4, NANOG, SOX2, and KLF4, as well as increased expression and activation of PPARγ receptors.

**Conclusions:**

All this data led us to suggest a clinical potential use of I_2_ for targeting CSC and improve current treatments against cervical cancer.

## Background

In 2012, cancer caused 8.2 million deaths with 14.1 million new cases with a higher prevalence in men than in women. Cervical cancer is the 4th most common cancer in women with an estimated of 528,000 new cases in 2012 and 266,000 deaths. According to the data, the mortality in Mexico for cancer in 2012 was 40,053, with cervical cancer responsible for 11.9% of those deaths [1].

High-risk human papillomaviruses (HPV) are related for the development of cervical cancer [2]. This is achieved through the persistent infection of HPV until later integrates his viral DNA into the host cell. The oncogenic potential of HPV resides in their oncoproteins E6 and E7 that disrupt the cell cycle control. The E6 function of high-risk HPV types is the binding and targeting of p53 for degradation and the function of E7 is binding the retinoblastoma tumor suppressor protein (pRb) for degradation allowing the release of the transcription factor (EF2) that promotes the expression of numerous genes that control DNA synthesis and cell proliferation [3, 4].

Tumors exhibit a high degree of cellular heterogeneity, and we now believe that only certain cells, known as cancer stem cells (CSC), have the ability to maintain the growth of the tumor mass and the capacity to invade other tissues. The proportion of CSC is variable depending on tumor type and stage [5, 6]. CSCs are undifferentiated cells that have the capacity for self-renewal, chemo-radiation resistance, promoting metastasis and cancer recurrence [7–9]. The presence of CSC has been shown in several types of cancers, such as breast, colon, brain and many others, as identified using the expression of various markers [10–14]. The presence of cervical cancer stem cells (CCSC) has been determined through the expression of CD49f and cytokeratin 17 (CK17) [15, 16].

CD49f, also known as alpha 6 integrin, belongs to the alpha family of integrins and is found on the cell membrane. CD49f has been used as an epithelial stem marker in the human epidermis [17], as an important marker for enrichment of cancer stem cells [18], and described as a target during HPV binding to initiate an intracellular signaling cascade for virus entry [19]. Cytokeratins are proteins that are part of epithelial cells, and their expression depends on the degree of differentiation they acquire. Keratin expression in cervical tissue has been well defined, and it was observed that sub-columnar reserve cells, where the stem cell population of the uterine cervix is found, showed specific expression of CK17 [20]. Identification and characterization of cancer stem cell-like cells from primary carcinomas of the cervix uteri found expression of CK17 [21], suggesting that cells expressing this cytokeratin also as a target of HPV [22]. Our group has previously shown expression of CD49f and CK17 as possible markers for CCSC and that sphere cultures (cervospheres) are enriched in CCSC showing high tumorigenic in vivo assays compared to their counterparts grown as monolayers [15, 16].

Self-renewal and pluripotency are maintained by the expression of Oct4, Sox2, Klf4, and Nanog on embryonic stem (ES) cells [23–26]. There is evidence that these stemness genes are relevant for tumor transformation, metastasis, and tumorigenicity in human malignancies [27–29]. In clinical terms, chemo and radio-therapy are the gold standard for the treatment of cervical cancer in advanced stages. Depending on the stage of the tumor, the rate of recurrent disease and inefficient treatments could be due to the presence of drug-resistant CSC. For these reasons, different investigations have focused on developing complementary approaches that involve natural compounds that decrease chemo-resistance and reduce the toxic effects of conventional therapies. I_2_ exerts significant antineoplastic effects on several types of cancer and multiple mechanisms could mediate its actions. Among these, I_2_ can react with arachidonic acid generating the iodolipid called 6-iodolactone (6-IL), which has been confirmed to be an agonist of the peroxisome proliferator-activated receptor type gamma (PPARγ). The activation of these receptors decreases the expression of specific markers associated with invasiveness and epithelial-mesenchymal transition [30–34]. Previous studies from our laboratory showed that I_2_ impairs chemo-resistance mechanisms, enhances doxorubicin retention and induces downregulation of chemo-resistance markers p21, Bcl-2 and MDR-1 in chemo-resistant MCF-7 cells [35]. In the present work, we showed that in cultures growing as monolayers or cervospheres of HeLa and SiHa cell lines derived from cervical cancer, I_2_ was able to inhibit proliferation and the ability to form tumors in mice. This effect includes a decrease in expression of CD49f and CK17 putative stem cell markers and stem transcription factors OCT-4, SOX2, KLF4, NANOG. We propose that in this model, the action of I_2_ could be through the activation of the PPAR gamma receptors.

## Methods

### Cell culture and I_2_

Human CC cell lines, HeLa (ATCC@-CRM-CCL-2 T, adenocarcinoma, HPV-18) and SiHa (ATCC@-HTB-35, squamous cell carcinoma, HPV-16), were obtained from ATCC (American Type Culture Collection, Manassas, VA, USA). Monolayer culture cells were seeded (500,000 cells) in 100 mm cell culture dishes (Corning, Inc., Corning, NY, USA) in 7 mL of medium, grown at 37 °C in a humidified atmosphere with 5% CO2 in DMEM media (Gibco®) supplemented with 10% fetal bovine serum (Gibco®) and 100 U/mL penicillin/streptomycin (Invitrogen, Carlsbad, CA, USA) Molecular iodine was prepared with 13 g of crystalline iodine (Macron-Avantor, Center Valley, PA, USA) and 60 g of potassium iodide (Sigma-Aldrich, St. Louis, MO, USA) in one liter of ddH_2_O. The iodine concentration was confirmed by titration with a solution of 0.1 N sodium thiosulfate. The use of all cell lines and cell cultures were approved by Research and Bioethics Committees of Instituto Nacional de Cancerología [Prot. No. 018/012/IBI) (CEI/1096/17), Dated 03.02.2018].

### Cervospheres

For the formation of cervospheres derived from CC cell lines, monolayer cell cultures were grown to 70–80% confluence and then harvested, counted, and washed with Phosphate buffer solution (PBS) to remove the remainder of FBS. After that, cells were cultured in DMEM F12 media (Gibco®) supplemented with 20 ng/mL epidermal growth factor (BioLegend Inc., San Diego, CA, USA), 20 ng/mL basic fibroblast growth factor (BioLegend Inc., San Diego, CA, USA), 10 μl/mL B27 (50X, Gibco®) and 100 U/mL penicillin/streptomycin (Invitrogen, Carlsbad, CA, USA) at a density of 3 × 10^3^ cells/ml in 100 mm ultra-low adherence dishes of (Corning, Inc., Corning, NY, USA). Cells were grown at 37 °C in a humidified atmosphere with 5% CO2 for 3 days and cervosphere formation was monitored daily.

### Molecular iodine treatments

The I_2_ was previously diluted in culture medium and then added to the cells. The monolayers and spheres cultures of SiHa and HeLa cell lines were treated at a concentration of 200 μM of I_2_ for 24 h. After time, cultures were collected and processed for the corresponding tests.

### Analysis of markers by flow cytometry

Monolayers and cervospheres treated or untreated with 200 μM of I_2_ for 24 h were collected separately and placed in a tube where they were allowed to remain for 15 min. After that time, supernatant was removed and the bottom cells were washed with PBS and spun down at 500 g (*r* = 11 cm) for 5 min at room temperature. Supernatant was removed and cells were resuspended in flow buffer (PBS 1X, 0.05% BSA) and disaggregated by mechanic pipetting. Before incubation with anti-CK17, anti-OCT-4, anti-SOX2, anti-NANOG, and anti-KLF4 antibodies, cells were permeabilized by incubation with methanol for 15 min on ice. Then, cells were washed and incubated with primary antibody. For each primary antibody, 5 × 10^5^ cells were incubated with anti-CD49f-PE (BD Bioscience, CA, USA), anti-CK17 (Santa Cruz Biotechnology, Inc., Dallas, TX, USA), anti-OCT-4-AlexaFluor488, anti-SOX2-AlexaFluor488 (both BioLegend Inc., San Diego, CA, USA), anti-NANOG-PE (BD Bioscience, CA, USA), anti-KLF4-APC (R&D Systems, Inc., Minneapolis, MN, USA) for 30 min on ice. After that time, cells were washed with 300 μL of flow buffer and spun down at 500 g (*r* = 11 cm) for 5 min at room temperature. Cells incubated for anti-CK17, after the time elapsed, were washed and incubated with FITC-coupled secondary antibody for 30 min on ice. At the end of the incubation, cells were washed again with flow buffer, spun down, and supernatant was removed. All cells were then washed and fixed with 4% p-formaldehyde in PBS. Every marker was also incubated with isotype controls under the same conditions of the primary antibodies. Stained cells were read in ATTUNE NXT (Thermo Fisher Scientific Inc). At least ten thousand events were recorded for each flow cytometry measurement. FlowJo® software was utilized for analyzing data.

### Western blot analysis

The total proteins from HeLa monolayers (confluence 70–80%) and cervospheres treated or untreated with 200 μM of I_2_ for 24 h were extracted with RIPA buffer (150 mM NaCl, 1% Nonidet P-40, 0.5% deoxycholate, 0.1% sodium dodecyl sulfate [SDS], and 50 mM Tris- HCl, pH 8.0) supplemented with a complete EDTA-free protease inhibitor cocktail (Roche, USA) and incubated on ice for 20 min. The protein concentrations were measured using the Bradford method (Bio-Rad, USA). Samples containing 70 mg of protein were boiled in SDS containing sample buffer (10% SDS, 20% glycerol, 20 mM Tris-Cl, pH 6.8, 10 mM β-mercapto-ethanol, and 0.05% bromophenol blue), separated by SDS polyacrylamide gel electrophoresis (SDS-PAGE) and transferred onto nitrocellulose membranes. The membranes were blocked with 5% nonfat milk in PBS containing 0.1% Tween-20 for 1 h and incubated with the appropriate antibody dilution. The PPARγ antibody (sc-7196, Santa Cruz Biotechnology, Inc., Dallas, TX, USA) was used at a dilution of 1:1000 and GAPDH antibody (sc-48167, Santa Cruz Biotechnology, Inc., Dallas, TX, USA) was used at a dilution of 1:10000. The following HRP-conjugated secondary antibodies were used at a dilution of 1:10000 anti-rabbit (sc-2313, Santa Cruz Biotechnology, Inc., Dallas, TX, USA) and the anti-goat (sc-2020, Santa Cruz Biotechnology, Inc., Dallas, TX, USA) was used at a dilution of 1:20000. The proteins were visualized through an enhanced chemiluminescence reaction using the Super Signals West Pico Chemiluminescent Substrate (Thermo Fisher Scientific Inc., Pierce Protein Research Products, Rockford, IL, USA). Densitometry was performed using ImageJ software (version 1.41, National Institutes of Health, Bethesda, MD, USA) and chemiluminescence was normalized to the level of GAPDH protein.

### Total RNA extraction, quantification of mRNA by real-time quantitative PCR (RT-qPCR)

RNA extraction of HeLa monolayers (confluence 70–80%) and cervospheres treated or untreated with 200 μM of I_2_ for 24 h was carried out using Trizol (Invitrogen, Cat. No. 15596026), the extracted RNA was treated with DNaseI (ThermoFisher, Cat. No. EN0521) and purified using the Direct-zol ™ kit, RNA MicroPrep (Zymo Research, Cat. No. R2060). The purified RNA was quantified using an Epoch ™ spectrophotometry system and subjected to retrotranscription with 2000 ng of RNA with the SuperScript™ IV First-Strand Synthesis System (Invitrogen, Cat. No. 18091050) to obtain cDNA. By means of real-time PCR, the cDNA was be evaluated to determine the expression levels of the PPARγ (F-PPARγ: TCT CTC CGT AAT GGA AGA CC and R-PPARγ: GCA TTA TGA GAC ATC CCC AC), PTEN (F-PTEN: GAT GAG GCA TTA TCC TGT ACA CA and R-PTEN: CTC TTC AGA TAC TCT TGT GCT GT) E6 (F-E6: GCG ACC CTA CAA GCT ACC TG and R-E6: GTT GGA GTC GTT CCT GTC GT) and E7 (F-E7: TGA AAT TCC GGT TGA CCT TC and R-E7: CAC GGA CAC ACA AAG GAC AG) genes. The oligonucleotides for the GAPDH gene (F-GAPDH: AAG GTC GGA GTC AAC GGA TTT G and R-GAPDH: CCA TGG GTG GAA TCA TAT TGG AA) were used as control. Placing 100 ng of cDNA, 12.5 μL of the master mix Maxima SYBR and 10 pmol of each oligonucleotide in a total volume of 25 μL carries out real-time PCR. The reaction conditions are 95 °C for 10 min for initial denaturation, 40 cycles of 95 °C for 15 s, 60 °C for 30 s and 72 °C for 30 s for denaturation, alignment and extension, respectively. The reaction was performed on the QIAGEN Rotor-Gene Q equipment. The expression levels of the mRNAs were determined from the threshold cycle (Ct), and the relative expression levels were calculated using the 2^-ΔΔCt^ method. For mRNA quantification, the Ct values were normalized to the expression of the GAPDH mRNA level.

### Viability assay

The effects in viability of HeLa and SiHa monolayers treated or untreated with 100, 200 and 400 μM of I_2_ for 24 h and HeLa cervospheres treated or untreated with 200 μM of I_2_ for 24 h, were analyzed using the OZBlue Cell Viability Kit (OZ Biosciences, San Diego, USA) following the supplier’s instructions.

### Animals and in vivo tumorigenic assays

This study was approved by Research and Bioethics of Instituto Nacional de Cancerología’s Committee [Prot. No. 018/012/IBI) (CEI/1096/17), Dated 03.02.2018]. NOD/SCID (Non-Obese Diabetic/Severe Combined Immunodeficiency) female mice were used in this work to test the tumorigenic capacity of CCSC-like I_2_-treated and untreated. The animals were 4–6 weeks old, weighed 21–25 g and were obtained from the Unidad de Modelos Biológicos of the Instituto de Investigaciones Biomédicas, UNAM. After acclimation in the bioterium of Instituto Nacional de Cancerología, they were randomly designated to experimental groups. Mice were xenotransplanted subcutaneously (s.c) with HeLa monolayer cells and their respective cervosphere cells treated or untreated with 200 μM of I_2_ for 24 h at different amounts using six mice per group. Throughout experiment, mice lived in a room with 12-h/12-h light/dark cycle, at a temperature of 27 °C, 60% of relative humidity and had free access to chow and water. Mice were monitored for at least 2 months. The handling and execution of experimental procedures in mice were carried out according with local and international guidelines such as the official Mexican standard NOM-062-ZOO-1999 (Technical specifications for the production, care and use of laboratory animals), the code of ethics of IIB–UNAM and other international guidelines. For subcutaneous tumors in mice, the maximal allowable size is 2 cm in diameter (Tumor Policy for Mice and Rats from Boston University Research Compliance). Tumor growth was monitored three times a week for up to 8 weeks. At the end of the 8 weeks, a humanitarian final point was made; the mice were euthanized in a compressed CO_2_ chamber. After that, tumors were extracted through a cut made in the skin and they were measured, weighed and photo-documented. Each tumor was measured by a vernier caliper and the tumor volume was calculated using the Attia-Weiss formula “Tumor volume = (0.4) (a) (b ^ 2)”, where “a” is the largest diameter and “b” the smallest diameter of each tumor.

### Statistical analysis

The data were analyzed using GraphPad Prism software (v 6.0; GraphPad Software, Inc., CA, USA). One or two-way ANOVA was performed to determine the significance of differences between groups. Data are expressed as mean ± standard deviation (SD), and values with *P* < 0.05 were considered statistically significant.

## Results

### Molecular iodine interferes with viability of HeLa and SiHa monolayer cell cultures and cervosphere formation of HeLa

**Fig. 1:**
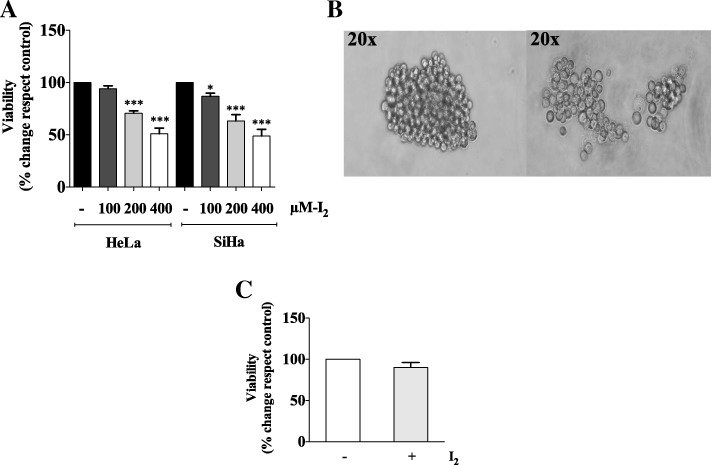
Effect of I_2_ on HeLa and SiHa cells and inhibition of HeLa cervosphere formation. (**a**) Viability assay of monolayer cultures of HeLa and SiHa cells treated with 100, 200 and 400 μM (I_2_) and deionized water (control) for 24 h. (**b**). HeLa cervospheres without treatment (left) and treated with 200 μM (I_2_) for 24 h (right) (**c**). HeLa cervosphere viability assay without treatment and treated with 200 μM (I_2_) for 24 h. Data are expressed as mean ± SD (*n* = 3 independent assays), and the asterisk indicates a significant difference with respect to the control (*P < 0.05, ***P < 0.001)

A dose-response curve (100 μM, 200 μM, 400 μM) of I_2_ for 24 h was carried out to evaluate cell viability under adherent (monolayer) culture conditions of HeLa and SiHa cells (Fig. 1a). HeLa cells were slightly more resistant than SiHa at 100uM I_2_ and the antiproliferative effect of I_2_ exhibited a sustained dose-response in both cells types. To evaluate a possible effect of I_2_ in CCSC-like cells, since 400 μM I_2_ showed high cytotoxicity, we selected 200 μM I_2_ for further experiments. A supplement of 200uM I_2_ (24 h) interfered with HeLa cervosphere formation, since the I_2_-treated cervospheres were observed to be smaller, with irregular conformation compared to the untreated cervospheres (Fig. 1b), however, cell viability wasn’t affected (Fig. 1c).

### CD49f expression in HeLa cervospheres and SiHa monolayer and cervosphere cultures is inhibited by molecular iodine

**Fig. 2:**
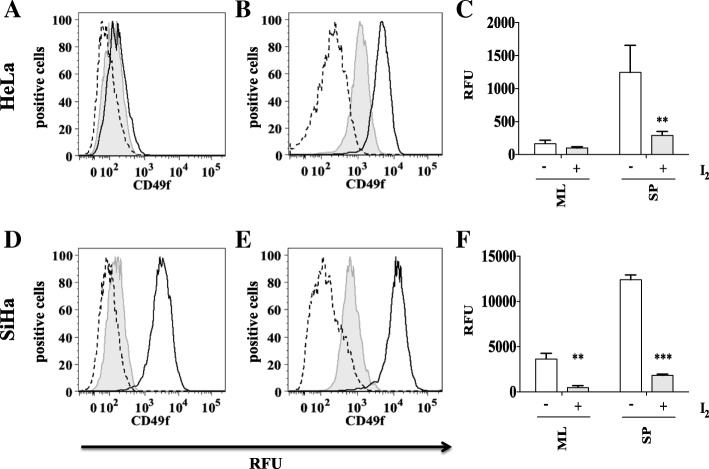
CD49f is reduced by I_2_ treatment in HeLa and SiHa monolayers and spheres cells. Monolayer cultures of HeLa and SiHa cells were treated with 200 μM (I_2_) for 24 h (tinted with line), without treatment (black line), isotype (long dashes) (**a** and **d**). Sphere cultures of HeLa and SiHa cells were treated with 200 μM of (I_2_) for 24 h (tinted grey with line), without treatment (black line), isotype (long dashes) (**b** and **e**). CD49f was analyzed by flow cytometry. (**a**, **b**, **d**, **e**). Data are expressed as mean ± SD (*n* = 3 independent assays), and the asterisk indicates a significant difference with respect to the control (**P < 0.01, ***P < 0.001) (**c** and **f**)

CD49f protein was evaluated under non-adherent conditions (cervospheres) for 7 days (data not shown). On the third day, we found the highest protein level of CD49f suggesting a significant proportion of cancer stem cell-like cells. Monolayer and cervospheres were treated on the second day with 200 μM I_2_ or deionized water (control) for 24 h. I_2_ was effective in reducing the expression of CD49f in HeLa and SiHa cervospheres and their respective monolayers, even the expression of CD49f in HeLa monolayer is very low, treatments with molecular iodine decreased its expression, although not to significant levels under these treatment conditions (Fig. 2).

### Molecular iodine significantly reduces CK17 in both HeLa and SiHa monolayer and cervosphere cultures

**Fig. 3:**
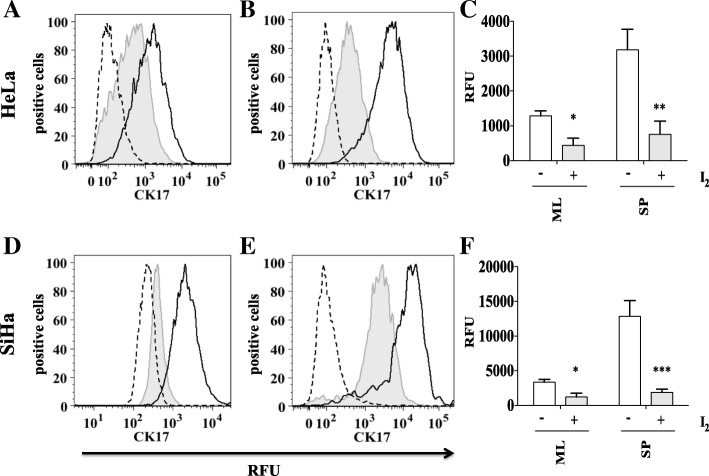
CK17 is reduced by I_2_ treatment in HeLa and SiHa monolayers and spheres cells. Monolayer cultures of HeLa and SiHa cells were treated with 200 μM (I_2_) for 24 h (tinted grey with line), without treatment (black line), isotype (long dashes) (**a** and **d**). Sphere cultures of HeLa and SiHa cells were treated with 200 μM (I_2_) for 24 h (tinted grey with line), without treatment (black line), isotype (long dashes) (**b** and **e**). CK17 was analyzed by flow cytometry. (**a**, **b**, **d**, **e**). Data are expressed as mean ± SD (n = 3 independent assays), and the asterisk indicates a significant difference with respect to the control (*P < 0.05, **P < 0.01, ***P < 0.001) (**c** and **f**)

CK17 is a marker for cervical stem cell identification [16, 21] and is an essential marker for the identification of CCSC. As with the CD49f molecular iodine assay, HeLa and SiHa cervospheres and monolayer cells were treated on the second day with 200 μM of I_2_ or deionized water (control) for 24 h. Cytometry analysis showed that I_2_ was capable of decreasing CK17 protein levels in both HeLa and SiHa cells under monolayer and cervosphere conditions (Fig. 3).

### OCT-4, SOX2, KLF4 and NANOG stemness markers are significantly reduced by molecular iodine treatment only in HeLa and SiHa cervospheres

**Fig. 4:**
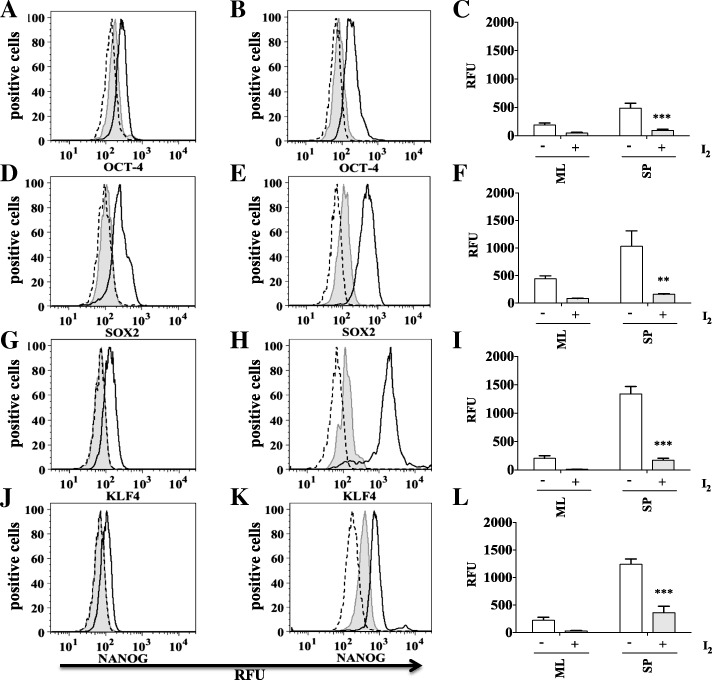
I_2_ treatment significantly reduces OCT-4, SOX2, KLF4 and NANOG in HeLa cells grown as spheres. Monolayer cultures of HeLa cells were treated with 200 μM (I_2_) for 24 h (tinted grey with line), without treatment (black line), isotype (long dashes) (**a**, **d**, **g**, **j**). Sphere cultures of HeLa cells were treated with 200 μM (I_2_) for 24 h (tinted grey with line), without treatment (black line), isotype (long dashes) (**b**, **e**, **h**, **k**). Markers were analyzed by flow cytometry. (**a**, **b**, **d**, **e**, **g**, **h**, **j**, **k**). Data are expressed as mean ± SD (*n* = 3 independent assays), and the asterisk indicates a significant difference with respect to the control (**P < 0.01, ***P < 0.001) (**c**, **f**, **i**, **l**)

**Fig. 5:**
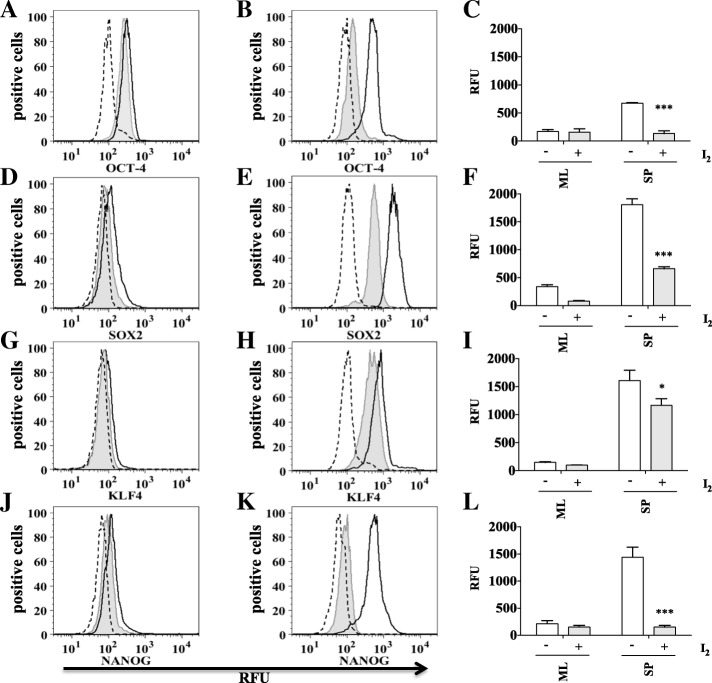
I_2_ treatment significantly reduces OCT-4, SOX2, KLF4 and NANOG in SiHa cells grown as spheres. Monolayer cultures of SiHa cells were treated with 200 μM (I_2_) for 24 h (tinted with line), without treatment (black line), isotype (long dashes) (**a**, **d**, **g**, **j**). Sphere cultures of SiHa cells were treated with 200 μM (I_2_) for 24 h (tinted with line), without treatment (black line), isotype (long dashes) (**b**, **e**, **h**, **k**). Markers were analyzed by flow cytometry (**a**, **b**, **d**, **e**, **g**, **h**, **j**, **k**). Data are expressed as mean ± SD (n = 3 independent assays), and the asterisk indicates a significant difference with respect to the control (*P < 0.05, ***P < 0.001) (**c**, **f**, **i**, **l**)

Identifying stemness markers such as OCT-4, SOX2, KLF4, and NANOG is needed to evaluate stemness of our cervospheres. We evaluated these transcription factors in HeLa and SiHa cells grown as monolayers and cervospheres in the presence or absence of I_2_ treatment. As expected, we found more stemness marker proteins in cervospheres compared to monolayer cells. Interestingly, OCT-4, SOX2, KLF4, and NANOG markers were down regulated by I_2_ treatments in both cervosphere and monolayer cultures of HeLa (Fig. 4) and SiHa (Fig. 5) cells.

### Activation of PPAR gamma is up-regulated by molecular iodine treatments in HeLa cells

**Fig. 6:**
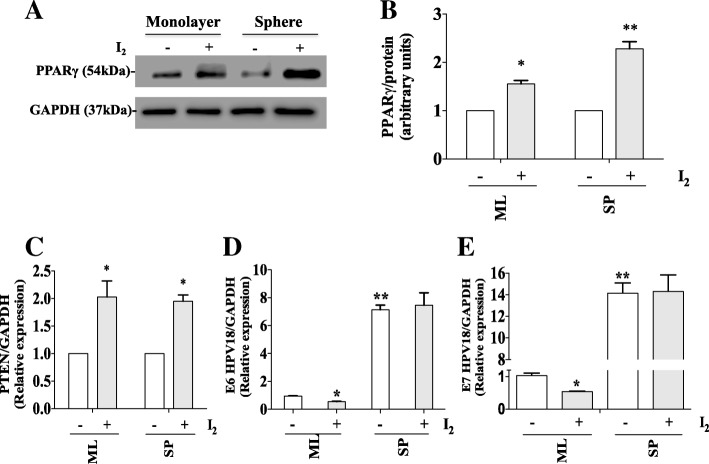
PPARγand PTEN are increased in HeLa cells with I_2_ treatment. Monolayer and sphere cultures of HeLa cells were treated with 200 μM (I_2_) for 24 h and PPARγ protein was quantified by Western blot and densitometry is reported as percent change with respect to control without treatment. (**a**, **b**). *PTEN* expression was analyzed by qPCR and normalized to *GAPDH* expression (**c**). HPV18 *E6* and *E7* oncoproteins expression were analyzed by qPCR and normalized to *GAPDH* expression (**d**, **e**). Data are expressed as mean ± SD (*n* = 3 independent assays), and the asterisk indicates a significant difference with respect to the control without treatment. (*P < 0.05, **P < 0.01)

It has been proposed that the antineoplastic effect of the molecular iodine is mediated by activation of PPARγ receptors by 6-iodolactone, in turn causing an increase in these receptors after treatment [33, 34]. Monolayer and cervospheres of HeLa cells were incubated with 200 μM of I_2_ for 24 h, resulting in a significant increase in PPAR gamma proteins compared to their untreated counterparts (Fig. 6a, b). To corroborate the activation of PPARγ we analyzed the expression of PTEN (phosphatase and tensin homolog deleted on chromosome ten), a known PPARγ-regulated gene (reviewed in [36]). Figure 6c shows that I_2_-supplemented HeLa cells exhibit significant increase of PTEN gene expression in both monolayers and cervospheres. This result indicates that the mechanism of the antineoplastic effects of molecular iodine could be through PPAR gamma. Figure 6d, e shows the effect of I_2_ on HPV18 *E6* and *E7* gene expression, resulting in a significant reduction of *E6* and *E7* expression in monolayers cells and no effect in cervospheres treated with I_2_.

### Molecular iodine treatments decrease the capacity for tumor formation of HeLa cervospheres

**Fig. 7:**
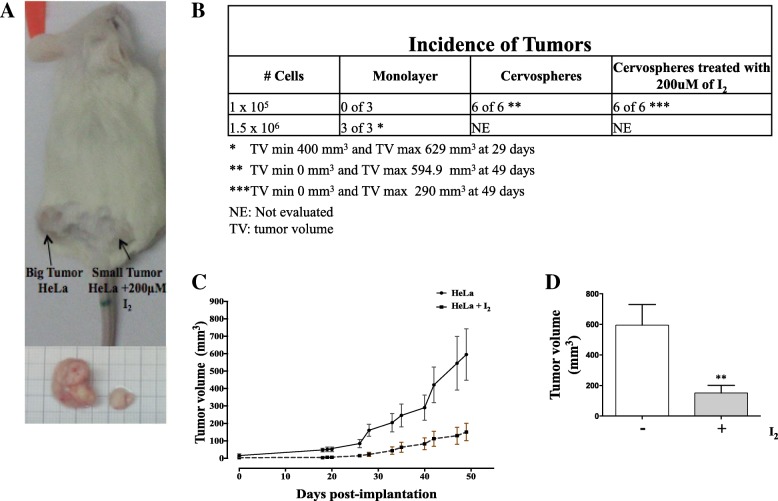
Effect of I_2_ on tumor growth in NOD/SCID mice. HeLa cervospheres were pre-incubated with 200 μM I_2_ or deionized water for 24 h. Each animal was inoculated with both subpopulations on each side (*n* = 6) and circles indicate sites of xenografts (**a**). Table showing the number of tumors developed in vivo (**b**). Average growth of tumor volume of xenograft tumors through the days (**c**). Average tumor volume size of cervospheres treated and untreated with I_2_ (**d**). Data are expressed as mean ± SD (*n* = 6 independent assays), and the asterisk indicates a significant difference with respect to the control (**P < 0.01) (**d**)

It has been demonstrated that cervospheres have higher tumorigenic capacity compared to their monolayer counterparts (15,16). In this paper, we evaluated the effect of I_2_ treatments on cervosphere tumorigenic capacity using an in vivo assay. Mice were inoculated with HeLa cervospheres pre-incubated for 24 h with 200 μM I_2_ or deionized water. Each animal was inoculated with both populations on the left or right side, respectively (Fig. 7a).

Figure 7b, c show that I_2_-treated cervospheres promoted smaller tumors in 6/6 mice, suggesting an anti-tumorigenic effect of I_2_ on these cervical cancer highly tumorigenic cells, as characterized by CD49f, CK17 and stemness markers. Tumors began to grow from 17 days after inoculation in the mice and tumor growth was evaluated for 49 days. We observed that untreated cervospheres formed bigger tumors with a maximum average size of 594.9 mm ^3^ whereas the cervospheres treated with I_2_ formed tumors of much smaller size, with a maximum average size of 150.8 mm^3^ (Fig. 7d). No adverse events were found in the experimental groups.

## Discussion

The percentage of cancer stem cells is very low in tumors, which makes it difficult to study them. Spheroidal cultures have been shown to enrich CSC-like cells and are a good system to evaluate CSC-related characteristics of solid tumors in vitro [37], but according to Blagosklonny (reviewed in [38]), these cells should be called stemloids since they possess high proliferation capacity, self-renewal and could be responsible for the reappearance of cancer after therapy. Many studies evaluate the biology of CSC and the mechanisms that give them chemo-resistance capacity. CSC shows resistance to many chemotherapeutics such as cisplatin, 5-FU, and doxorubicin. This is achieved through their high expression of pro-survival proteins, efficient ABC transporters to pump out drugs, signaling pathways that give them resistance properties, and much higher activated phosphorylation of DNA damage response factors [39–42]. Several strategies have been used to inhibit all these properties but they have not been enough, so the chemo-resistance of CSC requires new approaches aimed at eliminating these highly tumorigenic cells.

Molecular iodine has been studied in several cancer cell lines showing its ability to inhibit proliferation, chemo-resistance, and apoptotic effects [30–35], however there are no reports on its effect on cervical cancer cell lines nor on cultures enriched with cancer stem-like cells. HeLa and SiHa are the most representative cervical cancer cell lines and in this study, we used cultures grown under non adherent conditions (cervospheres) where we obtained a higher proportion of CSCC-like cells compared to traditional monolayer cultures, allowing us to study the effect of I_2_ on CSC derived from cervical cancer cell lines. We showed that I_2_ treatment decreased cervosphere formation, additionally, we observed living cells and some dead cells in these spheres; however, we do not know whether I_2_ treatment induces death in CCSC-like cells. Our group, as well as other authors, has proposed CD49f and CK17 as putative markers to isolate cervical cancer stem cells [15, 16]. CD49f is considered to be a stem cell marker for normal and cancer cells and is the only marker that is shared by more than 30 stem cell populations, being one of its main characteristics the maintenance of self-renewal (reviewed in [43]). It’s also a crucial molecule for the growth and survival of the breast stem cell-like subpopulation that displays increased proliferation and greater resistance to pro-apoptotic agents [44]. The importance of CD49f in cervical cancer is enhanced by its ability to act as a co-receptor for the entry of HPV into the host cells [45, 46]. CK17 is a marker of the HPV target cell, the cervical reserve (stem) cell which gives rise to metaplasia, and loss of CK17 induces cellular differentiation and attenuates tumorigenesis in cervical epithelia [21, 22, 47, 48]. We observed that cells treated with I_2_ show a decrease in CD49f and CK17 protein expression, molecules considered to be CCSC phenotype markers (reviewed in [43, 49]). Since CD49f is important for maintaining stem cell self-renewal, we hypothesize that the decrease in CD49f + cells caused by I_2_ could have an important effect on the CCSC-like biology, including tumorigenic capability. To validate the tumorigenic capability of a human cancer cell line, xenotransplantation must be performed in immunodeficient mice, a model used to promote tumor growth derived from human cancer for the analysis of malignant tumors and the evaluation of antitumor drugs [50]. Since I_2_ exhibited similar effects on HeLa and SiHa cell lines, we decided to use only HeLa cells for in vivo experiments to evaluate its effect over CCSC. Mice assays, using I_2_-treated cells, we observed that I_2_ treatment delays tumor growth, and make them unable to keep the tumor growing, suggesting that I_2_-treated CCSC-like cells have a lower capacity for tumor formation compared to non-treated CCSC-like cells. However, since the cells are still alive after I_2_ treatment, it’s clear that I_2_ treatment can help reduce their tumorigenic capability as observed through the reduction of tumor size and delayed tumor growth. Evaluation of HeLa monolayer cells treated with I_2_ were not pursued, since we focused our interests in evaluating the effect of I_2_ in cancer stem-like cells. Additionally, the effect of I_2_ on CCSC-like cells was observed by evaluating stemness markers, such as NANOG, SOX2, KLF4 and OCT-4, in cervospheres. It has been demonstrated that there is an increase in expression of these stemness markers in cervical carcinomas, compared with normal cervical tissue, and that their overexpression in cell lines derived from cervical cancer confers them an increased capacity for proliferation, clonogenicity, and tumorigenicity in vitro and in vivo, as well as promoting stem cell characteristics [51–54]. Interestingly, we observed different stemness marker levels between HeLa and SiHa cervospheres, mainly in KLF4 and SOX2 protein levels. This indicates that the mechanisms for self-renewal could be cell type-dependent. Our cervospheres showed overexpression of these stemness markers compared with their monolayer counterparts, as reported [15, 16]. Furthermore, I_2_ had the ability to decrease their expression in cervospheres, supporting a role for I_2_ in decreasing the tumorigenic capacity of these cancer cells, because as reported in the literature, the decrease of these stem cell markers makes the cancer cells less tumorigenic [27–29, 54]. The mechanism proposed by which I_2_ decreases cell proliferation is through the interaction and activation of the PPAR gamma receptor [34, 55]. In this work we show that in CCSC-like cells derived from I_2_-treated cervical cancer cell lines, PPAR gamma protein level was increased compared to untreated CCSC-like cells. As consequence, PPAR gamma activation promotes greater expression of its target gene, PTEN [56], in our CCSC-like cultures compared to their monolayer counterparts. These observations suggest that the mechanism whereby I_2_ decreases CD49f, CK17 and stemness markers in CCSC-like cells could be mediated by the activation of PPAR gamma receptors and consequently the activation of its PTEN response gene involved in self-renewal mechanisms. In addition to decreasing self-renewal capacity, PTEN activation reduces cell proliferation and tumorigenicity in CSC (reviewed in [57]). However, additional assays are needed to further address the mechanism by which I_2_ is able to decrease the tumor volume derived from CCSC-like cells.

Tyagi and collaborators demonstrated that the increase of E6 and E7 gene expression is close related with the stemness mediated by HES1, a specific transcription factor of NOTCH signal pathway [58]. In our conditions, we also observed an increase of E6/E7 expression in spheres cultures. Interesting, I_2_ treatment decrease the E6/E7 expression in HeLa monolayer cell culture conditions, an effect that was no observed in sphere culture conditions, suggesting that I_2_ doesn’t have any effect on E6/E7 HPV18 gene expression in CCSC-like.

## Conclusions

In resume, our results demonstrate the I_2_-mediated cytotoxic effect in CSC derived from cervical cancer cell lines, in which CD49f, CK17 and stemness marker positive cells are decreased. Since I_2_ supplements are considered to be safe for the treatment of diseases such as human mammary fibrocystic disease, breast cancer or prostate hyperplasia (reviewed in [30]), we suggest that I_2_ treatment for cancer should be studied in preclinical trials to evaluate its potential anti-cancer effect alone or in a combination with conventional therapeutic drugs, to eliminate cancer stem cells from cervical cancer and others.

## Abbreviations

6-IL: 6-iodolactone
CCSC: Cervical cancer stem cells
CSC: Cancer stem cells
ES: Embryonic stem
HPV: Human papillomaviruses
I_2_: Molecular Iodine
PPARγ: Peroxisome proliferator-activated receptor type gamma
PTEN: Phosphatase and tensin homolog deleted on chromosome ten
